# Reducing False Positives in Newborn Screening: The Role of Perinatal Factors in the Dutch NBS Program

**DOI:** 10.3390/metabo15090634

**Published:** 2025-09-22

**Authors:** Nils W. F. Meijer, Rose E. Maase, Patricia L. Hall, Wouter F. Visser, Klaas Koop, Annet M. Bosch, M. Rebecca Heiner-Fokkema, Monique G. M. de Sain-van der Velden, the CLIR-NBS Group

**Affiliations:** 1 Section Metabolic Diagnostics, Department of Genetics, University Medical Center Utrecht, Lundlaan 6, 3584 EA Utrecht, The Netherlands; 2 Reference Laboratory for Neonatal Screening, Center for Health Protection, National Institute for Public Health and the Environment (RIVM), 3720 BA Bilthoven, The Netherlands; 3 Department of Laboratory Medicine and Pathology, Mayo Clinic, Rochester, MN 55905, USA; 4 Amsterdam Gastroenterology Endocrinology and Metabolism, Division of Metabolic Diseases, Department of Pediatrics, Emma Children’s Hospital, Amsterdam UMC, University of Amsterdam, Meibergdreef 9, 1105 AZ Amsterdam, The Netherlands; 5 Laboratory of Metabolic Diseases, Department of Laboratory Medicine, University Medical Center Groningen, University of Groningen, 9700 RB Groningen, The Netherlands

**Keywords:** birthweight, gestational age, age at heel prick, biological sex, biomarkers, positive predictive value, newborn screening

## Abstract

**Background/Objectives**: Dutch newborn screening is an important public health program designed to detect conditions early in life, enabling timely interventions that can prevent mortality, morbidity, and long-term disabilities. However, the program also faces certain challenges. One such issue is obtaining and maintaining a high positive predictive value (PPV); another is that newborn screening (NBS) in the Netherlands is intended for all newborn babies until the age of six months. This means comparing infants at different ages may introduce variability that complicates data interpretation. To support the optimization of the program, we systematically analyzed population-level tandem mass spectrometry (MS/MS) data to explore postnatal metabolic changes. **Methods**: We evaluated the impact of covariates—including birth weight, gestational age, age at blood collection, and biological sex—on metabolite profiles using retrospective newborn screening (NBS) data. Special emphasis was placed on the combined effects of these covariates. The analysis was based on data from 985,629 newborns collected between 2018 and 2024. **Results**: Specifically, (extremely) preterm infants exhibit altered levels of several amino acids and acylcarnitines. Moreover, we observed multiplicative effects of gestational age and birth weight on several metabolic markers. Biological sex however, does not have an impact. The largest impact of the age of sampling was observed on the C0/C16+C18 ratio, which may impact screening performance for CPT1 deficiency. **Conclusions**: Covariate-adjusted reference values could improve the performance of the Dutch newborn screening.

## 1. Introduction

Newborn screening (NBS) programs are effective public health initiatives that help reduce morbidity and prevent mortality in individuals with inherited genetic disorders and other treatable conditions that can be identified at birth. Through timely intervention, the Dutch NBS program has averted negative outcomes for many newborns, highlighting its importance as a public health prevention strategy [1]. The Dutch NBS program includes 27 congenital disorders, with additional conditions under consideration or awaiting implementation [2].

In NBS, the concentration of informative (bio)markers extracted from heel prick blood is assessed against a pre-defined cut-off value to determine whether a neonate is at increased risk for certain disorders. Although the NBS program is highly effective at identifying positive cases in this way, the positive predictive value (PPV) for most inborn metabolic disorders (IMD) remains relatively low as a result of false positive referrals [1]. With the availability of new effective treatments and screening tests, there is additional justification for the expansion of the NBS program [3,4,5]. The number of false-positive test results may further increase, and with it, the associated burden on families and the healthcare system as indirect costs associated with unnecessary clinical follow-up increase [6,7]. Critical evaluation of screening data could help reduce false-positive rates for many disorders by improving test specificity, paving the way for future program expansions while reinforcing the role of NBS as an effective public health prevention strategy.

Covariates such as birthweight, gestational age, age at heel prick and biological sex are known to impact the measured concentration of informative biomarkers and therefore can influence diagnostic accuracy if they are not corrected for. For example, several studies have shown that biological sex may influence biomarkers [8]. Furthermore, preterm birth is considered one of the most significant risk factors contributing to false-positive as well as false-negative results. Moreover, false-negative outcomes may occur in preterm newborns as the flux through immature biosynthetic pathways may not yet be sufficient to result in an elevation of the biomarkers of interest [9,10,11,12]. Various studies have investigated the effect of sampling age on NBS markers, other than those from MS/MS [13,14,15,16]. However, from the very few studies that have explored the effect on MS/MS markers, for the sampling age significant changes in markers have been observed, especially when the heel prick is obtained after 30 days of age [10,12]. Previous studies have identified the effects of individual covariates on biomarker concentration, but few focus on the combined effect of these factors, taking into consideration multiplicative effects on markers contributing to false-positive or false-negative outcomes. Collaborative Laboratory Integrated Reports (CLIR) is an interactive Web Tool with clinical utility that has been used to improve the performance of newborn screening by integrating covariates and focusing on disease range comparisons [17].

In the current study, we examined the impact of birthweight, gestational age, age at heel prick, biological sex, and combinations thereof on all tandem mass spectrometry (MS/MS) markers currently used in the Dutch NBS program to determine whether these factors should be taken into consideration when interpreting screening results. Additionally, we compared our findings to the moving percentiles observed in CLIR to assess their applicability to other screening programs.

## 2. Materials and Methods

### 2.1. The Study Population

Data over the period from 1 January 2018 to 31 December 2024 were extracted retrospectively from the registry used by the Dutch National Institute for Public Health and the Environment, Department for Vaccine Supply and Prevention Programs (RIVM-DVP). Each dataset comprised the following elements: biological sex, gestational age, age at heel prick and the concentration of all markers included in the NeoBase2^TM^ Kit from Revvity (Turku, Finland). A list of all included markers is provided in Supplementary Table S1. Prior to data analysis, the data underwent pseudonymization. Permission for use of the data was given by the commission Data applications Praeventis of the RIVM-DVP. Data were included only from neonates whose parents did not object (before 2023) or consented (since 2023) to the use of heel prick data for research purposes.

### 2.2. Data Selection and Inclusion

The study population included samples derived from neonates between 3 and 183 days of age. The target age of the Dutch NBS is between 72 and 168 h (3–7 days). A small subset of neonates (0.078%) was screened prior to 72 h (3 days) of age, which is earlier than the target screening moment and therefore may have included severely ill newborns. To avoid inclusion of data that limit the generalizability of the conclusions, these samples were excluded from the dataset. In the Netherlands, NBS is offered to children up to the age of 6 months; for this reason, the upper age limit for inclusion in this study was set to 183 days. Neonates with birthweight in the range 500–6000 g were included in the study, as were neonates with a gestational age of 161 to 308 days (23–43 weeks). Datasets derived from neonates identified as positive during screening and referred for further clinical assessment as a result were not included in the study.

### 2.3. Data Analysis

Prior to analysis of each marker, rows containing a zero value (indicating an inconclusive measurement) were removed. Next, values above the 99th percentile and below the 1st percentile for the marker of interest were excluded to minimize the impact of outliers. Following outlier removal, data were categorized into gestational age groups according to the following criteria: Extreme premature (EP) (<28 weeks), Premature (P) (28≤ to <32 weeks), Late premature (LP) (32≤ to <37 weeks), Term (T) (37≤ to ≤42 weeks) and Post term (POS) (>42 weeks). Each gestational age group was further subdivided into three weight-for-gestational-age categories: small for gestational age (SGA: below the 10th percentile), average for gestational age (AGA: 10th to 90th percentile), and large for gestational age (LGA: above the 90th percentile). This resulted in the following categories: SGA EP, AGA EP, LGA EP, SGA P, AGA P, LGA P, SGA LP, AGA LP, LGA LP, SGA T, AGA T, LGA T, SGA POS, AGA POS, and LGA POS categories. An overview of the median, mean, count, and z-score for each of these categories is displayed in Supplementary Table S2.

For age at heel prick, the same process for removing zero values and outliers was applied. Subsequently, the data were categorized based on the day the heel prick was performed relative to birth, resulting in the following groups: day 3, day 4, day 5, day 6, day 7, days 8–14, and days 15–183. An overview of the median, mean, count, and z-score for each of the age groups is displayed in Supplementary Table S3. The categories were defined based on the NBS target age (3–7 days) as well as two extended time intervals intended to capture potential early and later changes in marker levels. The latter two categories were selected to include sufficient and approximately the same number of cases.

Following stratification, data were normalized by calculating z-scores for each marker by subtracting the study population mean from the group mean and dividing the outcome by the study population standard deviation. (i.e., (average (SGA EP free carnitine)—average (study population free carnitine))/standard deviation (study population free carnitine)). Additionally, we divided the largest observed deviation (ΔC) by the cut-off values currently used in the Dutch NBS (CO) of the respective marker and multiplied this value by 100 (deviation/cut-off value × 100 = Δ%) to determine the relevance of the observed change in concentration. The deviation observed between the population mean and the group that differs most from it is the number selected as the largest observed deviation (ΔC). With regard to the ΔC, a 10% deviation was selected as the threshold to identify meaningful changes in a marker. This approach was taken as small standard deviations (indicating a small spread in the data) may result in large z-scores even though the change in concentration is relatively small. Z-scores for each marker were subsequently depicted in a heatmap. To compare our results to the moving percentiles in CLIR, we utilized the marker versus covariate plot from the productivity tools in CLIR.

## 3. Results

### 3.1. Characteristics of the Study Population

The current study included 985.629 datasets comprising analytical data from the Dutch NBS program. In total, 21 markers derived from the NeoBase2^TM^ kit from Revvity were assessed (Supplementary Table S1). These markers were selected based on their current use in the characterization of IMDs within the Dutch NBS program [18]. Demographic characteristics of the study population are shown in Table 1. The population consisted of 51.27% males and 48.73% females. Within this cohort, 6.38% were born preterm (<37 weeks), 93.23% were born at term (37 to 42 weeks), and 0.39% were post-term (>42 weeks). The median age at which the heel prick was performed was 4 days, with 975.731 (99.00%) samples within the target window (72–168 h) and 9898 (1.00%) sampled after 1 week of age. Birthweight was normally distributed with 0.81% having a low birth weight (<1500 g) and 12.94% had a birthweight above 4000 g (Table 1).

### 3.2. The Combined Impact of Gestational Age and Birthweight on Dutch NBS Markers

**Figure 1:**
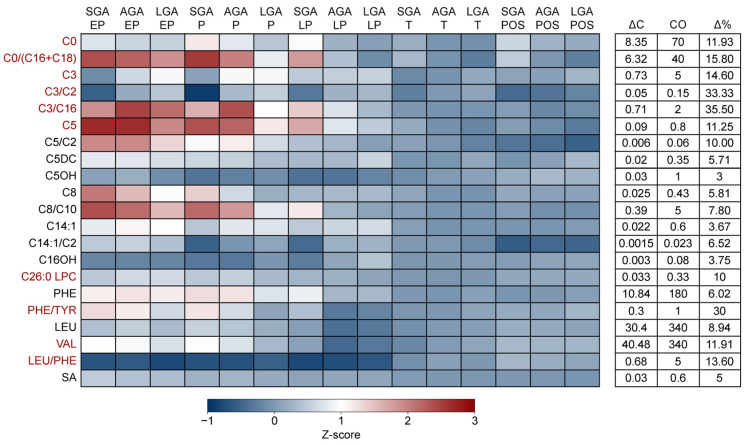
**The impact of gestational age and birthweight on markers in the Dutch NBS.** Heatmap and table depicting the concentration (z-score), difference in concentration between the group with the lowest and highest mean value (ΔC), cut-off value (CO) and percentual concentration difference relative to the cut-off value (Δ%) of NBS markers for different combinations of gestational age and birthweight. Markers for which the Δ% was high > 10% are colored red. Z-scores were calculated by subtracting the study population mean from the group mean and dividing the outcome by the study population standard deviation. (i.e., (average (SGA EP C0)—average (study population C0))/standard deviation (study population C0)). The Δ% was calculated by dividing ΔC by the cut-off value (CO) and multiplying the outcome by 100 (ΔC/CO × 100). Abbreviations: SGA—small for gestational age, AGA—average for gestational age, LGA—large for gestational age, EP—extremely preterm, P—preterm, LP—late preterm, T—Term, POS—post-term. An overview of the data for each group can be found in Supplementary Table S2.

There is an inherent positive correlation between gestational age and birthweight, which can confound interpretations of how gestational age affects biomarker levels [9,10,11,12]. We therefore investigated the combined effect of gestational age and birthweight on the levels of all markers currently included in the Dutch NBS program as measured by MS/MS (Figure 1). Biological sex was excluded from the final analysis, as none of the markers significantly differed between male and female neonates suggesting that biological sex was unlikely to confound or modify the outcome. (Supplementary Figure S1). Among the 21 metabolites and ratios studied, we observed an increase of C0, the C0/C16+18 ratio, C3, the C3/2 ratio, the C3/C16 ratio, C5, C26:0 LPC, the phenylalanine/tyrosine ratio and valine relative to the cut-off (>10 Δ%) in the blood spots derived from preterm neonates compared to term neonates. Overall, a larger increase in concentration was observed in neonates born prior to 32 weeks (EP and P groups) in comparison to those born between 32 and 37 weeks (LP group). In contrast, the leucine/phenylalanine ratio was reduced in preterm neonates. Free carnitine levels were elevated in post-term newborns compared to term newborns, with the largest effect being observed in babies with a low birthweight relative to their gestational age (SGA). A similar trend was observed for the C0/C16+18 ratio; however, here a high birthweight relative to GA (LGA) fully negated the effects of post-term birth. The negative association between birthweight and marker concentration was also observed for C5 and the C0/C16+C18 ratio, while birthweight positively affected the levels of C3 and the C3/C2 ratio. Overall, these results indicate that the combined effects of either SGA or LGA and preterm birth may influence screening outcome for Carnitine Palmitoyltransferase 1 (CPT1) deficiency, Isovaleric acidemia (IVA), Phenylketonuria (PKU), Methylmalonic Acidemia (MMA), Propionic acidemia (PA), Adrenoleukodystrophy (ALD), and Maple syrup urine disease (MSUD) and should be taken into consideration when screening for these disorders.

### 3.3. The Impact of Age of Sampling on NBS Marker Levels

**Figure 2:**
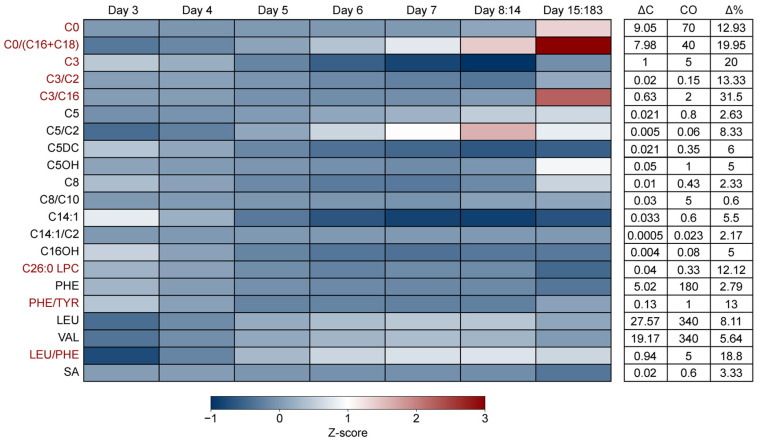
**The impact of the age of sampling on markers in the Dutch NBS.** Heatmap depicting the concentration (z-score), change in concentration between the group with the lowest and highest mean value (ΔC), cut-off value (CO) and percentual concentration change relative to the cut-off value (Δ%) of NBS markers for different age categories. Markers for which the Δ% was high > 10% are colored red. Z-scores were calculated by subtracting the study population mean from the group mean and dividing the outcome by the study population standard deviation. (i.e., (average (SGA EP C0)—average (study population C0))/standard deviation (study population C0)). The Δ% was calculated by dividing ΔC by the cut-off value (CO) and multiplying the outcome by 100 (ΔC/CO × 100). An overview of the data for each group can be found in Supplementary Table S3.

Although the target screening moment for NBS is 72–168 h (3–7 days) after birth, NBS sampling may occur at a later stage in development, up to six months of age. Consequently, the effects of preterm birth may decrease as development progresses, while the impact of food derived metabolites and endogenous synthesis will increase. To address this, we examined the effect of age across the first 6 months after birth (Figure 2). In line with previous studies, we found a large impact of age at sampling on the C0/C16+C18 ratio, which showed a strong increase relative to the cut-off (>10 Δ%) from day 8 onwards [12]. A similar effect was observed for C0, the C3/C16 ratio and the leucine/phenylalanine ratio, although the increase began at later ages. In contrast, C26:0 LPC decreased slightly beyond day 14. Interestingly, C3, the C3/C2 ratio and the phenylalanine/tyrosine ratio initially decreased but started to normalize after 14 days. Comparing our results to the moving percentiles observed in CLIR revealed a similar trend, with the exception of C26:0 LPC, indicating that these patterns are consistent with data from other NBS programs (Supplementary Figure S2–S9).

## 4. Discussion

The Dutch NBS program is continually being reevaluated to ensure it remains an effective preventive health strategy, incorporating the latest developments in the field. Alongside refinement of cut-offs based on additional insights from true and false positive referrals, this may also include amendment of the screening algorithm to include additional biochemical and/or sequencing tiers to improve program performance. As novel technologies emerge, screening methods become more sensitive and specific, resulting in the ability to identify neonates at higher risk of disease more accurately and to develop screening programs for new disorders [19,20]. For example, maleylacetoacetate isomerase deficiency (MAAI-D) has recently been identified as a distinct biochemical variant, also characterized by hypersuccinylacetonemia, similar to hepatorenal tyrosinemia, and which can result in false positive screening results for Tyrosinemia type 1 [19]. Furthermore, development and reimbursement of innovative therapies for rare diseases, which need to be administered pre-symptomatically, may in the coming years increase the number of diseases for which NBS is necessary to ensure timely detection for treatment to be effective [3]. These key factors are driving the considerations regarding the future expansion of the Dutch NBS program. To accommodate this growth, it is essential to reduce the false positive referral rate and associated patient and family burden and healthcare costs.

In the current study, we examined the impact of covariates (gestational age, birthweight, age at heel prick and biological sex on all markers included in the Dutch NBS program measured by MS/MS to define whether adjusting for these factors could help to improve the PPV. We demonstrate that the concentrations of carnitine, C3, C5, C26:0 LPC, phenylalanine, leucine, valine and the ratios C0/(C16+C18, C5/C2, C3/C16 and the phenylalanine/tyrosine are increased in preterm neonates. Although C8, C14:1 and the C8/10 ratio were also affected, their relatively minor concentration differences may be less relevant from an NBS perspective. Additionally, we observed multiplicative effects of gestational age and birthweight on several markers. Specifically, these effects were seen for C0 (in the context of SGA and prematurity), the C0/(C16+C18) ratio (SGA and prematurity), C3 (LGA and prematurity), C5 (SGA and prematurity), and the phenylalanine/tyrosine ratio (SGA and prematurity).

Whilst the majority of markers and corresponding cut-off values are considered ‘primary markers’ and are used in the NBS decision-making process, two ratios, C5/C2 and C8/C10, are considered ‘Secondary markers’ for IVA and MCADD, respectively. As secondary markers, these ratios are monitored for potential clinical utility but are to date not validated as informative for the Dutch NBS program. The results from our study show that, as for C5, the C5/C2 decreases with gestational age. Based on this information, the screening for IVA may be improved by using a higher cut-off limit for C5 and C5/C2 in preterm neonates as compared to term and post-term neonates. However, before redefining such a cut-off, this finding should be validated using positive controls from a patient cohort. Furthermore, it is recommended that the consideration, validation, and subsequent inclusion of C5/C2 as an informative marker for IVA take into account demographic variability to ensure reliable assessment.

In the Dutch NBS, CPT1 deficiency is identified by evaluation of both an elevated C0 concentration and an elevated C0/(C16+C18) ratio. Moreover, primary carnitine deficiency (PCD), which is currently an incidental finding of the screening, but will soon be implemented as a new target disease in the Netherlands [2], is detected by a low C0 concentration. In line with previous findings, our results, i.e., higher C0 and C0/(C16+C18) ratio in preterm children, emphasize that screening for these disorders may be improved by adjusting cut-off values for gestational age and birthweight [12]. However, before redefining any cut-off values, this finding should be validated in a patient cohort. Moreover, our data demonstrate that the age at which the heel prick test is performed can significantly affect these markers. This is particularly relevant for neonates screened at 8 days of age or later—outside the target NBS window—and applies to populations such as expatriates returning to the Netherlands and refugees. Our data may help explain the recent rise in false-positive referrals for CPT1 deficiency, which has been observed and tentatively linked to the inclusion of older children in the Dutch NBS program of late [1]. The extent to which gestational age and birthweight continue to influence these markers later in infancy remains unclear. However, it is likely that other factors, which are introduced after birth, may attenuate or negate the influence of the formerly mentioned cofactors. This is emphasized by the finding that carnitine levels in premature infants normalize to term infant levels 6 days after birth [10].

A similar impact of gestational age was observed for C3, C5, C26:0 LPC, phenylalanine, leucine and valine, which are some of the primary markers for PA/MMA, IVA, ALD, PKU and MSUD, respectively. The fact that birthweight may attenuate or even negate the effects of preterm birth on marker levels, as can be seen for C3 in our results, reinforces the idea that multivariate adjustment may be needed to obtain accurate screening outcomes. Indeed, previous studies have shown that the integration of covariate adjusted cut-offs and integration with post-analytical tools may reduce the number of second tier tests required. The data from this study suggest that for conditions such as CPT1 deficiency, PCD, PA and MMA, the use of CLIR may offer an effective approach for simultaneously adjusting marker concentrations for multiple covariate effects and this warrants further investigation.

## 5. Conclusions

For some disorders, it may be possible to realize improvement in NBS performance by implementation of an alternative cut-off value for a particular marker or marker ratio for a particular situation, such as prematurity. However, for other disorders, improvements in NBS are likely only achievable by simultaneously accounting for multiple covariate effects on marker concentrations. This necessitates the use of CLIR or, alternatively, machine learning models/custom regression-based adjustment models. While several logistical and legal barriers must be overcome to integrate CLIR into routine practices of NBS programs, the results from the current study indicate that covariate-adjusted reference ranges may be the most effective way to improve the accuracy of the NBS.

## 6. Limitations and Benefits of the Study

The use of a large dataset comprising data over multiple years offers a significant advantage, as it enhances the reliability and allows for more robust statistical analyses across diverse population subgroups. Furthermore, it allows the analysis of multiplicative effects between covariates on markers. Despite these advantages, the following limitation must be considered: the current results reflect the impact of covariates on markers within the reference population; however, these effects may differ in the condition-specific population and thus must be evaluated prior to redefining screening algorithms.

## Figures and Tables

**Table 1:** Demographics of the Dutch NBS study population from 2018 to 2024.

Category	Count (Percentage)
Biological sex	
Male	505,322 (51.27%)
Female	480,307 (48.73%)
Total	985,629 (100%)
Birthweight in grams	
<1000	2691 (0.27%)
1000–1499	5273 (0.53%)
1500–1999	11,024 (1.12%)
2000–2499	35,032 (3.55%)
2500–2999	131,040 (13.30%)
3000–3499	339,973 (34.49%)
3500–4000	333,044 (33.79%)
>4000	127,552 (12.94%)
Total	985,629 (100%)
Age at heel prick in days	
3	107,297 (10.89%)
4	474,684 (48.16%)
5	221,651 (22.49%)
6	147,899 (15.01%)
7	24,200 (2.46%)
8–14	4678 (0.47%)
>14	5220 (0.53%)
Total	985,629 (100%)
Gestational age in weeks	
<28	2595 (0.26%)
28–31	6588 (0.67%)
32–37	53,731 (5.45%)
37–42	918,919 (93.23%)
>42	3796 (0.39%)
Total	985,629 (100%)

## Data Availability

The datasets presented in this article are not readily available because the data are part of an ongoing study. Requests to access the datasets should be directed to the corresponding author.

## Supplementary Materials

The following supporting information can be downloaded at: https://www.mdpi.com/article/10.3390/metabo15090634/s1, Table S1: Marker abbreviations; Table S2: The impact of gestational age and birthweight on markers in the Dutch NBS; Table S3: The impact of age on markers in the Dutch NBS; Figure S1: The impact of biological sex on markers in the Dutch NBS; Figures S2–S9: Moving percentiles derived from CLIR, indicating the impact of age on carnitine, C0/(C16+C18), C3, C3/C2, C3/C16, C26:0 LPC, PHE/TYR, and LEU/PHE in newborn infants.

## Abbreviations

The following abbreviations are used in this manuscript:

NBS	Newborn screening
PPV	Positive Predictive Value
MS/MS	Tandem mass spectrometry
IMD	Inborn metabolic disorder
CLIR	Collaborative Laboratory Integrated Reports
EP	Extreme premature
P	Premature
LP	Late premature
T	Term
POS	Post term
SGA	Small for gestational age
AGA	Average for gestational age
LGA	Large for gestational age
ΔC	Largest observed deviation
CO	Cut-off value
Δ%	The relevance of the observed change in concentration
CPT1	Carnitine Palmitoyltransferase 1
IVA	Isovaleric acidemia
PKU	Phenylketonuria
MMA	Methylmalonic acidaemia
ALD	Adrenoleukodystrophy
MSUD	Maple syrup urine disease
MAAI-D	Maleylacetoacetate isomerase deficiency
MCADD	Medium-chain acyl-CoA dehydrogenase deficiency
PCD	Primary carnitine deficiency
PA	Propionic acidemia
