# Supplementary material for: Reducing False Positives in Newborn Screening: The Role of Perinatal Factors in the Dutch NBS Program
Source: Metabolites. 2025 Sep 22;15(9):634. doi: 10.3390/metabo15090634 (PMC12471867; doi:10.3390/metabo15090634)
Supplement: Supplementary file 1 [file metabolites-15-00634-s001.zip › metabolites-3824417-supplementary.pdf]

Supplementary information

Table S1. Marker abbreviations

Code	Explanation
C0	Free Carnitine
C0/(C16+C18)	Acetylcarnitine / (Palmitoylcarnitine + Oleoylcarnitine) ratio
C3	Propionylcarnitine
C3/C2	Propionylcarnitine / Acetylcarnitine ratio
C3/C16	Propionylcarnitine / Palmitoylcarnitine ratio
C5	Isovalerylcarnitine
C5/C2 (*)	Isovalerylcarnitine / Acetylcarnitine ratio
C5DC	Glutarylcarnitine
C5OH	3-hydroxyisovalerylcarnitine
C8	Octanoylcarnitine
C8/C10 (*)	Octanoylcarnitine / Decanoylcarnitine ratio
C14:1	Tetradecenoylcarnitine
C14:1/C2	Tetradecenoylcarnitine / Acetylcarnitine ratio
C16:OH	3-Hydroxypalmitoylcarnitine
C26:0 LPC	C26:0-lysophosphatidylcholine
PHE	Phenylalanine
PHE/TYR	Phenylalanine / Tyrosine ratio
LEU	Leucine
VAL	Valine
LEU/PHE	Leucine / Phenylalanine ratio
SA	Succinylacetone

Table S2. The impact of gestational age and birthweight on markers in the Dutch NBS.
Table displaying the median (M - µmol/l), mean (µmol/l), count (N) and z-score of NBS markers for different combinations of gestational age and birthweight. Z-scores were calculated by subtracting the study population mean from the group mean and dividing the outcome by the study population standard deviation. (i.e. (average(SGA EP C0) - average (study population C0)) / standard deviation (study population C0)).
(*): C5/C2 and C8/C10 are considered 'Secondary markers' for IVA and MCADD respectively. As secondary markers these ratios are monitored for potential clinical utility but are to date not validated as informative for the NBS.

	SGA EP	AGA EP	LGA EP	SGA P	AGA P	LGA P	SGA LP	AGA LP	LGA LP	SGA T	AGA T	LGA T	SGA POS	AGA POS	LGA POS
**C0 M**	23.12	21.08	21.22	25.78	22.41	20.27	24.64	18.91	17.41	18.7	17.16	16.91	21.52	19.04	18.65
**Mean**	23.37	22.49	22.18	26.37	23.68	21.54	25.44	20.18	18.91	19.95	18.26	18.02	22.51	20.27	19.73
**N**	214	1846	226	593	4772	589	5079	41511	5112	89819	722337	90186	368	2967	369
**Z-score**	0.72	0.59	0.54	1.18	0.77	0.45	1.04	0.24	0.05	0.21	-0.05	-0.08	0.59	0.26	0.17
**C0/(C16+C18) M**	10.91	10.53	9.79	11.37	9.88	6.93	9.34	5.88	4.72	5.74	4.95	4.57	6.19	5.09	4.63
**Mean**	10.87	10.5	9.74	11.23	9.97	7.34	9.52	6.36	5.18	6.19	5.35	4.91	6.61	5.44	4.94
**N**	172	1502	183	533	4316	526	5174	41485	5174	90262	722574	90273	374	2989	373
**Z-score**	2.38	2.22	1.88	2.54	1.99	0.82	1.79	0.39	-0.14	0.31	-0.06	-0.25	0.5	-0.02	-0.24
**C3 M**	1.49	2.03	2.28	1.65	2.28	2.28	1.94	1.97	1.98	1.51	1.58	1.71	1.71	1.65	1.78
**Mean**	1.65	2.16	2.37	1.79	2.37	2.36	2.06	2.09	2.1	1.64	1.7	1.84	1.8	1.78	1.92
**N**	233	1973	222	590	4793	585	5138	41214	5147	90249	722019	90234	370	2982	367
**Z-score**	-0.13	0.62	0.93	0.08	0.93	0.92	0.48	0.62	0.54	-0.14	-0.05	0.15	0.1	0.07	0.28
**C3/C2 M**	0.08	0.11	0.12	0.07	0.12	0.13	0.09	0.12	0.12	0.1	0.11	0.11	0.09	0.09	0.1
**Mean**	0.09	0.12	0.13	0.08	0.12	0.13	0.1	0.12	0.12	0.1	0.11	0.12	0.1	0.1	0.11
**N**	246	2021	231	603	4966	608	5134	41215	5093	89453	723132	89518	370	2954	370
**Z-score**	-0.55	0.17	0.45	-0.90	0.33	0.52	-0.32	0.24	0.29	-0.24	0.004	0.1	-0.44	-0.35	--0.19
**C3/C16 M**	1.09	1.35	1.28	1.08	1.31	0.86	0.99	0.78	0.68	0.59	0.58	0.59	0.6	0.56	0.55
**Mean**	1.15	1.32	1.23	1.09	1.29	0.92	1.03	0.84	0.74	0.65	0.63	0.64	0.66	0.61	0.62
**N**	132	1097	136	412	3402	417	5088	41117	5132	90471	724838	89954	369	2986	368
**Z-score**	1.86	2.48	2.15	1.62	2.37	1.02	1.4	0.71	0.32	-0.02	-0.06	-0.04	0.04	-0.16	-0.11
**C5 M**	0.17	0.17	0.15	0.16	0.15	0.12	0.13	0.1	0.09	0.08	0.08	0.07	0.08	0.07	0.07
**Mean**	0.17	0.17	0.14	0.16	0.15	0.12	0.14	0.11	0.1	0.09	0.08	0.08	0.09	0.08	0.08
**N**	141	1149	143	507	4063	507	5126	41369	5158	90921	727525	90758	372	3000	372

	SGA EP	AGA EP	LGA EP	SGA P	AGA P	LGA P	SGA LP	AGA LP	LGA LP	SGA T	AGA T	LGA T	SGA POS	AGA POS	LGA POS
Z-score	2.66	2.65	1.93	2.35	2.21	1.18	1.67	0.75	0.5	0.19	-0.08	-0.18	0.04	-0.13	-0.28
C5/C2 M	0.01	0.011	0.009	0.008	0.008	0.007	0.006	0.006	0.005	0.005	0.005	0.005	0.004	0.004	0.004
Mean	0.01	0.01	0.009	0.008	0.009	0.007	0.007	0.006	0.006	0.006	0.006	0.005	0.005	0.005	0.004
N	186	1535	190	606	4985	621	5246	42098	5227	90123	721362	90112	362	2907	358
Z-score	1.92	1.91	1.34	0.94	1.16	0.66	0.39	0.29	0.06	0.09	-0.02	-0.21	-0.34	-0.4	-0.55
C5DC M	0.07	0.07	0.07	0.07	0.07	0.07	0.06	0.07	0.07	0.06	0.06	0.06	0.06	0.06	0.07
Mean	0.08	0.08	0.07	0.07	0.07	0.07	0.07	0.07	0.07	0.06	0.06	0.06	0.06	0.06	0.07
N	235	1980	227	636	5096	629	5272	42230	5278	90768	726997	90807	375	3002	370
Z-score	0.77	0.76	0.69	0.6	0.53	0.36	0.32	0.32	0.56	-0.03	-0.04	0.13	-0.05	0.08	0.3
C5OH M	0.19	0.19	0.18	0.16	0.17	0.18	0.16	0.16	0.17	0.17	0.18	0.18	0.19	0.2	0.19
Mean	0.19	0.20	0.18	0.17	0.17	0.18	0.17	0.17	0.18	0.18	0.19	0.19	0.2	0.2	0.2
N	231	2038	233	644	5155	641	5249	42220	5241	90574	725505	90024	372	2984	369
Z-score	0.08	0.22	-0.1	-0.36	-0.22	-0.09	-0.38	-0.35	-0.19	-0.11	0.03	0.09	0.19	0.31	0.26
C8 M	0.05	0.05	0.04	0.04	0.04	0.03	0.03	0.03	0.03	0.03	0.03	0.03	0.03	0.03	0.03
Mean	0.055	0.049	0.042	0.047	0.038	0.033	0.034	0.034	0.035	0.032	0.031	0.03	0.033	0.032	0.032
N	196	1644	203	600	4842	604	5259	42705	5274	91361	731230	91386	375	3019	373
Z-score	2.06	1.55	0.97	1.38	0.63	0.22	0.28	0.3	0.32	0.11	-0.04	-0.06	0.16	0.09	0.08
C8/C10 M	1	1	1	1	1	0.75	0.8	0.67	0.67	0.67	0.67	0.67	0.67	0.63	0.67
Mean	1.03	0.99	0.9	1	0.94	0.78	0.84	0.7	0.68	0.66	0.65	0.65	0.66	0.65	0.64
N	126	1071	133	470	3823	477	5223	41821	5222	91278	731644	91390	376	3038	374
Z-score	2.36	2.15	1.56	2.17	1.8	0.77	1.18	0.27	0.11	0.04	-0.04	-0.03	0.03	-0.08	-0.09
C14:1 M	0.07	0.08	0.07	0.06	0.07	0.07	0.06	0.07	0.07	0.05	0.05	0.05	0.05	0.05	0.05
Mean	0.073	0.078	0.077	0.066	0.072	0.071	0.067	0.069	0.07	0.058	0.056	0.056	0.056	0.057	0.058
N	225	1864	213	606	5019	620	5236	42013	5247	90299	723472	89755	372	2980	366
Z-score	0.8	1.08	1.01	0.44	0.75	0.72	0.48	0.6	0.65	0.06	-0.05	-0.04	-0.04	-0.03	0.03
C14:1/C2 M	0.0041	0.0043	0.0042	0.0029	0.0035	0.0037	0.003	0.0039	0.0039	0.0036	0.0036	0.0034	0.0028	0.003	0.0029
Mean	0.0044	0.0045	0.0042	0.0031	0.0037	0.0039	0.0032	0.0041	0.0041	0.0038	0.0038	0.0036	0.003	0.0032	0.0032
N	236	1983	230	623	5104	633	5143	41895	5183	89526	722008	89698	366	2950	366
Z-score	0.48	0.56	0.38	-0.53	-0.04	0.12	-0.44	0.23	0.25	0.00	0.00	-0.13	-0.59	-0.46	-0.49

		SGA EP	AGA EP	LGA EP	SGA P	AGA P	LGA P	SGA LP	AGA LP	LGA LP	SGA T	AGA T	LGA T	SGA POS	AGA POS	LGA POS
C16OH	M	0.01	0.01	0.01	0.01	0.01	0.01	0.01	0.01	0.01	0.01	0.01	0.01	0.01	0.01	0.01
	Mean	0.012	0.011	0.011	0.011	0.011	0.012	0.011	0.013	0.014	0.012	0.012	0.013	0.013	0.013	0.013
	N	238	2029	232	635	5080	635	5265	42148	5233	88946	714191	89169	372	2980	368
	Z-score	-0.18	-0.19	-0.19	-0.33	-0.32	-0.04	-0.27	0.12	0.41	-0.03	-0.01	0.04	0.04	0.1	0.12
C26:0 LPC	M	0.16	0.17	0.16	0.15	0.16	0.15	0.14	0.14	0.14	0.13	0.13	0.13	0.14	0.14	0.14
	Mean	0.16	0.17	0.17	0.16	0.17	0.15	0.15	0.15	0.14	0.14	0.14	0.14	0.14	0.15	0.15
	N	233	1996	232	628	5072	630	5202	41936	5193	90128	722156	89572	370	2984	370
	Z-score	0.47	0.63	0.63	0.47	0.53	0.32	0.28	0.23	0.10	0.01	-0.02	-0.01	0.13	0.14	0.17
PHE	M	52.37	53.545	52.85	54.93	53.465	49.23	49.915	44.88	45.02	42.21	42.01	42.93	41.72	42.135	43.01
	Mean	52.53	53.29	52.72	53.71	53.28	49.26	50.20	45.64	45.80	43.11	42.87	43.77	42.88	43.11	43.73
	N	211	1770	206	557	4500	555	5112	41431	5171	90105	722876	89703	369	2984	373
	Z-score	1.13	1.22	1.16	1.27	1.22	0.73	0.85	0.29	0.31	-0.02	-0.04	0.07	-0.04	-0.01	0.06
PHE/TYR	M	0.88	0.82	0.71	0.86	0.70	0.57	0.64	0.51	0.54	0.57	0.58	0.60	0.62	0.60	0.63
	Mean	0.84	0.82	0.72	0.83	0.72	0.62	0.67	0.54	0.56	0.60	0.60	0.62	0.63	0.62	0.65
	N	176	1406	174	491	3983	489	4886	39369	4880	90002	726207	90099	375	3002	371
	Z-score	1.29	1.15	0.63	1.21	0.64	0.11	0.38	-0.32	-0.20	-0.02	0.00	0.11	0.18	0.13	0.27
LEU	M	150.05	154.63	147.155	156.78	151.44	142.49	137.27	122.08	125.86	132.4	136.2	137.46	141.445	140.64	138.58
	Mean	151.48	156.00	149.78	157.05	153.20	146.11	141.24	126.65	129.57	136.57	140.08	141.12	145.59	144.51	142.74
	N	223	1963	232	629	5031	629	5126	41134	5137	90099	722315	89667	370	2993	366
	Z-score	0.38	0.53	0.33	0.56	0.44	0.21	0.06	-0.40	-0.31	-0.09	0.02	0.06	0.20	0.16	0.11
VAL	M	155.53	153.73	147.69	163.30	147.17	130.91	125.66	111.59	116.31	120.59	125.84	128.46	135.55	132.97	132.37
	Mean	155.40	153.68	147.11	156.23	147.65	136.43	130.79	115.75	119.70	124.05	128.98	131.45	136.45	135.83	135.57
	N	184	1601	198	534	4372	545	5017	40224	4971	90212	724535	89796	370	2997	369
	Z-score	1.00	0.93	0.69	1.03	0.71	0.30	0.09	-0.46	-0.32	-0.16	0.02	0.11	0.30	0.28	0.27
LEU/PHE	M	2.81	2.83	2.72	2.78	2.76	2.88	2.70	2.72	2.78	3.14	3.24	3.19	3.35	3.32	3.26
	Mean	2.84	2.87	2.77	2.82	2.81	2.91	2.77	2.80	2.86	3.21	3.30	3.26	3.45	3.39	3.34
	N	229	1989	230	619	4963	606	5011	40284	5029	90004	723235	89991	370	2984	367
	Z-score	-0.66	-0.62	-0.78	-0.69	-0.70	-0.54	-0.78	-0.72	-0.63	-0.08	0.07	0.00	0.29	0.20	0.12
SA	M	0.13	0.13	0.12	0.12	0.12	0.11	0.11	0.11	0.11	0.1	0.1	0.1	0.1	0.1	0.1

	SGA EP	AGA EP	LGA EP	SGA P	AGA P	LGA P	SGA LP	AGA LP	LGA LP	SGA T	AGA T	LGA T	SGA POS	AGA POS	LGA POS
Mean	0.14	0.14	0.13	0.13	0.13	0.12	0.12	0.12	0.12	0.11	0.11	0.11	0.11	0.11	0.11
N	239	2064	236	645	5184	641	5255	42247	5220	90219	722767	90279	370	2972	371
Z-score	0.49	0.40	0.37	0.26	0.28	0.18	0.11	0.13	0.11	0.02	-0.01	-0.03	-0.01	-0.02	-0.04

Table S3. The impact of age on markers in the Dutch NBS.
Table displaying the median (M - µmol/l), mean (µmol/l), count (N) and z-score of NBS markers for different age categories. Z-scores were calculated by subtracting the study population mean from the group mean and dividing the outcome by the study population standard deviation. (i.e. (average(Day 3 C0) - average (study population C0)) / standard deviation (study population C0)).

	Day 3	Day 4	Day 5	Day 6	Day 7	Day 8:14	Day 15:183
C0 M	17.35	17.26	17.55	17.43	17.65	18.37	27.27
Mean	18.65	18.45	18.65	18.48	18.64	19.33	27.50
N	104625	464431	218093	145867	23871	4555	4546
Z-score	0.012	-0.019	0.012	-0.014	0.011	0.114	1.349
C0/(C16+C18) M	4.35	4.70	5.35	6.05	6.88	8.39	13.22
Mean	4.76	5.12	5.72	6.41	7.24	8.68	12.74
N	104759	466419	219421	146444	23787	4115	965
Z-score	-0.32	-0.16	0.10	0.41	0.78	1.41	3.21
C3 M	1.93	1.76	1.49	1.26	1.10	0.96	1.51
Mean	2.05	1.88	1.60	1.35	1.18	1.05	1.67
N	104382	467308	219077	144241	22744	3664	4700
Z-score	0.46	0.21	-0.20	-0.56	-0.81	-1.00	-0.10
C3/C2 M	0.11	0.11	0.11	0.10	0.10	0.09	0.11
Mean	0.11	0.11	0.11	0.11	0.10	0.10	0.12
N	104542	464697	217754	145576	23731	4527	5087
Z-score	0.06	0.06	-0.04	-0.14	-0.23	-0.34	0.17
C3/C16 M	0.60	0.60	0.58	0.57	0.57	0.59	1.28
Mean	0.66	0.66	0.63	0.62	0.62	0.66	1.25
N	104932	466111	218008	145259	23710	4511	3388
Z-score	0.05	0.04	-0.06	-0.11	-0.11	0.03	2.24

		Day 3	Day 4	Day 5	Day 6	Day 7	Day 8:14	Day 15:183
C5	M	0.08	0.08	0.08	0.08	0.09	0.09	0.1
	Mean	0.083	0.083	0.086	0.089	0.092	0.100	0.104
	N	104945	466773	219162	146609	23958	4588	5076
	Z-score	-0.08	-0.05	0.02	0.13	0.24	0.50	0.62
C5/C2	M	0.0043	0.0048	0.0056	0.0068	0.0079	0.0096	0.0071
	Mean	0.0047	0.0052	0.0061	0.0072	0.0082	0.0097	0.0078
	N	103934	466645	219071	144728	23111	3890	4539
	Z-score	-0.42	-0.23	0.14	0.59	1.02	1.62	0.82
C5DC	M	0.07	0.06	0.06	0.05	0.05	0.05	0.05
	Mean	0.070	0.064	0.058	0.054	0.052	0.049	0.051
	N	105108	469352	219675	146300	23894	4527	5046
	Z-score	0.44	0.14	-0.17	-0.38	-0.48	-0.63	-0.55
C5OH	M	0.19	0.18	0.18	0.18	0.17	0.18	0.23
	Mean	0.19	0.19	0.18	0.18	0.18	0.18	0.23
	N	105697	468363	219060	146187	23878	4598	3697
	Z-score	0.11	0.02	-0.03	-0.09	-0.13	-0.04	0.91
C8	M	0.03	0.03	0.03	0.03	0.03	0.03	0.03
	Mean	0.035	0.032	0.029	0.028	0.027	0.029	0.037
	N	105918	471008	220759	147470	24136	4643	5137
	Z-score	0.35	0.09	-0.15	-0.29	-0.31	-0.15	0.56
C8 /C10	M	0.67	0.67	0.67	0.67	0.67	0.67	0.67
	Mean	0.66	0.66	0.66	0.65	0.65	0.67	0.68
	N	106157	469278	220092	147062	24062	4618	5197
	Z-score	0.01	0.02	-0.02	-0.02	-0.05	0.08	0.12
C14:1	M	0.07	0.06	0.05	0.04	0.04	0.04	0.04
	Mean	0.073	0.062	0.051	0.044	0.041	0.040	0.044
	N	103770	469657	219369	143766	22739	4185	4801
	Z-score	0.80	0.22	-0.31	-0.65	-0.83	-0.87	-0.67
C14:1/C2	M	0.0039	0.0036	0.0034	0.0034	0.0035	0.0039	0.0033

	Day 3	Day 4	Day 5	Day 6	Day 7	Day 8:14	Day 15:183
Mean	0.0041	0.0038	0.0036	0.0036	0.0037	0.0041	0.0036
N	104232	465351	217788	145415	23768	4505	4885
Z-score	0.29	0.03	-0.12	-0.13	-0.08	0.28	-0.16
C16OH M	0.01	0.01	0.01	0.01	0.01	0.01	0.01
Mean	0.015	0.013	0.011	0.011	0.011	0.011	0.011
N	105732	467298	214317	139358	22324	4157	4335
Z-score	0.56	0.09	-0.20	-0.34	-0.38	-0.32	-0.30
C26:0 LPC M	0.15	0.14	0.13	0.12	0.13	0.12	0.11
Mean	0.15	0.14	0.13	0.13	0.13	0.13	0.11
N	104263	465811	217390	145762	23790	4574	5112
Z-score	0.26	0.08	-0.12	-0.21	-0.13	-0.13	-0.47
PHE M	44.73	42.63	41.57	41.05	41.03	40.785	39.17
Mean	45.52	43.55	42.51	41.98	41.94	41.96	40.50
N	105266	465393	217330	144879	23732	4544	4779
Z-score	0.28	0.04	-0.09	-0.15	-0.16	-0.15	-0.33
PHE/TYR M	0.67	0.59	0.54	0.54	0.54	0.53	0.60
Mean	0.68	0.61	0.56	0.56	0.56	0.55	0.62
N	104730	464004	217663	145874	23902	4554	5183
Z-score	0.43	0.07	-0.19	-0.19	-0.23	-0.25	0.08
LEU M	122.33	131.44	141.66	147.58	151.175	151	141
Mean	126.23	135.22	145.10	150.62	153.80	153.52	143.57
N	104240	466500	217544	144537	23586	4508	4999
Z-score	-0.42	-0.13	0.18	0.36	0.46	0.45	0.13
VAL M	115.09	122.44	129.58	133.02	135.005	132.775	126.24
Mean	118.72	125.59	132.52	135.90	137.89	135.61	129.18
N	103506	465484	218225	145395	23786	4546	4983
Z-score	-0.35	-0.10	0.15	0.28	0.35	0.27	0.03
LEU/PHE M	2.73	3.07	3.40	3.59	3.68	3.70	3.62
Mean	2.78	3.13	3.45	3.63	3.71	3.72	3.65

		Day 3	Day 4	Day 5	Day 6	Day 7	Day 8:14	Day 15:183
	N	103691	467658	217754	143831	23377	4495	5105
	Z-score	-0.75	-0.20	0.30	0.58	0.70	0.73	0.61
SA	M	0.11	0.1	0.1	0.1	0.09	0.09	0.08
	Mean	0.11	0.11	0.11	0.11	0.11	0.10	0.09
	N	106329	467723	217456	144304	23266	4527	5104
	Z-score	0.04	0.03	-0.02	-0.07	-0.09	-0.12	-0.34

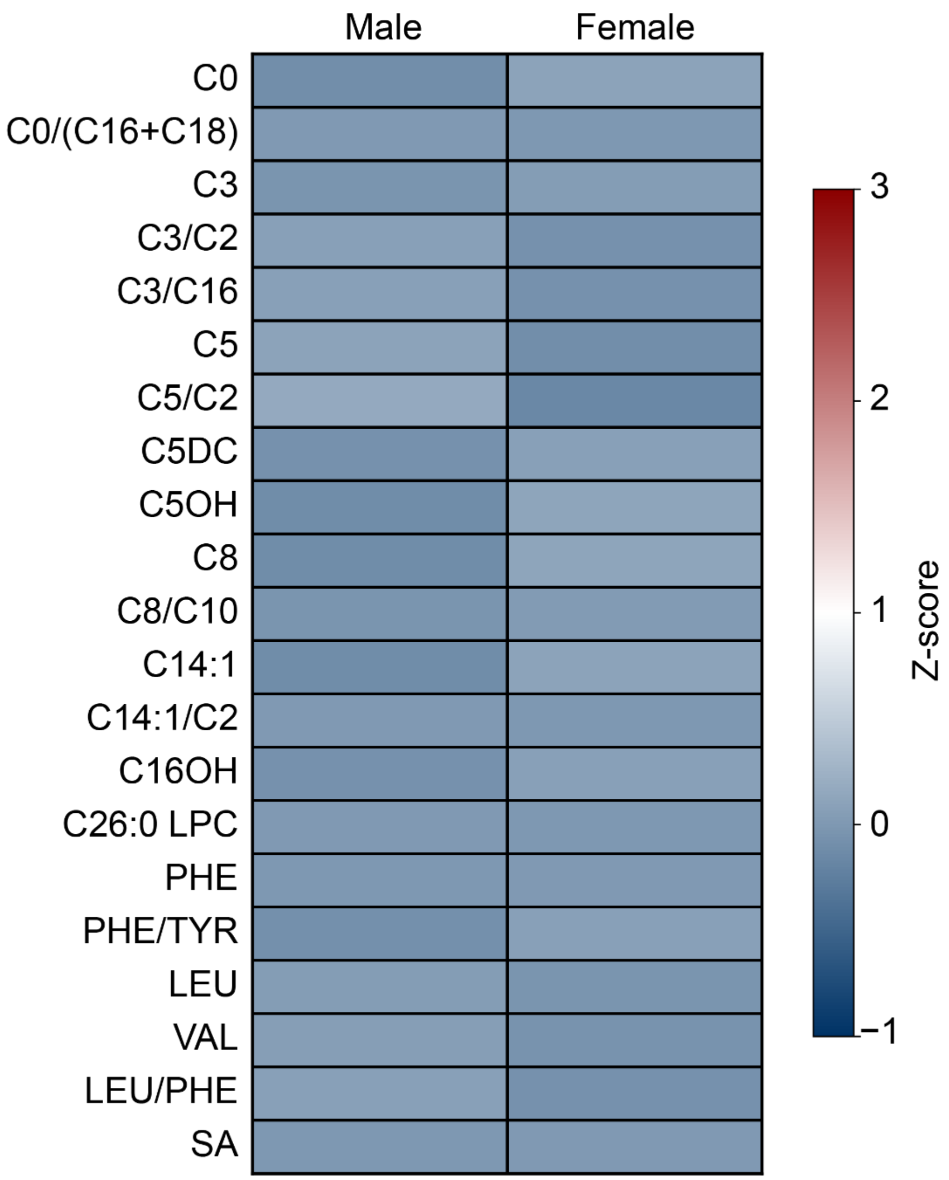

Figure S1. The impact of biological sex on markers in the Dutch NBS.
Heatmap depicting the concentration (z-score) of NBS markers for male and female neonates. Z-scores were calculated by subtracting the study population mean from the group mean and dividing the outcome by the study population standard deviation. (I.E. (average(Male C0) - average (study population C0)) / standard deviation (study population C0)).

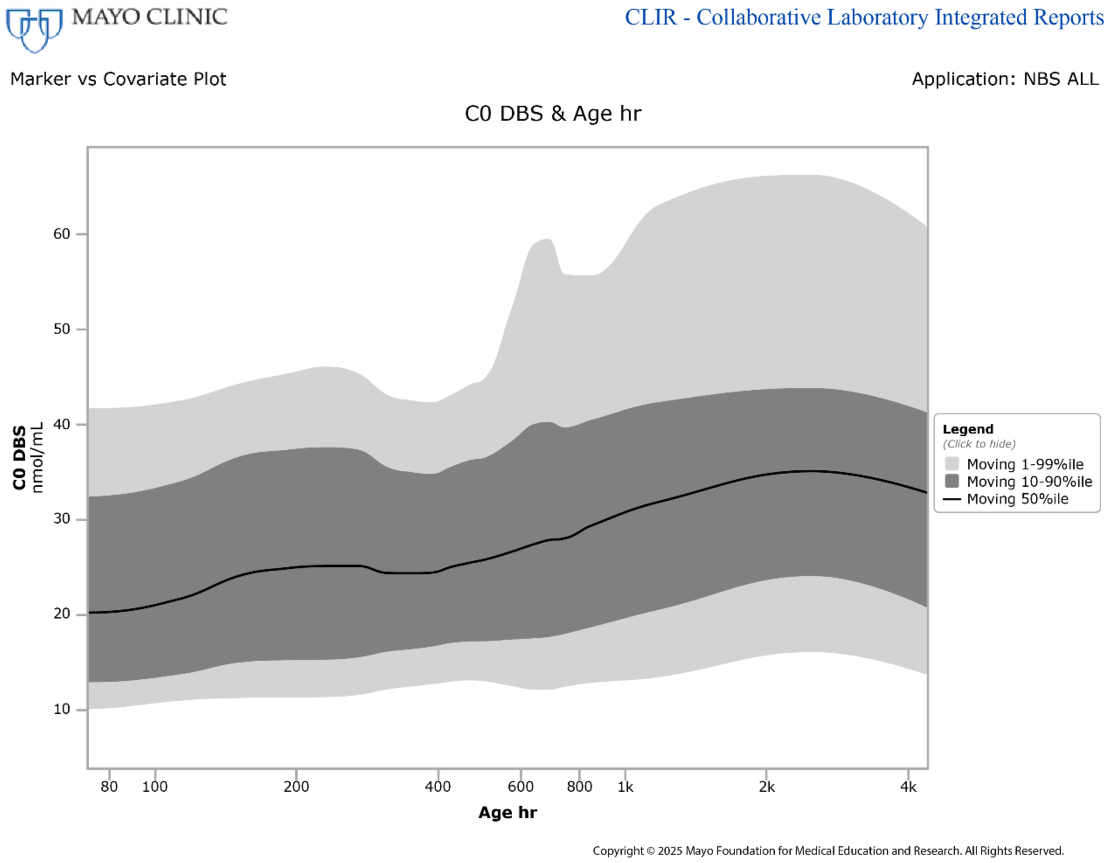

Figure S2. Moving percentiles derived from CLIR, indicating the impact of age on free carnitine levels (nmol/ml) in newborn infants. Retrieved from https://clir.mayo.edu/ (accessed on the 17th of June 2025).

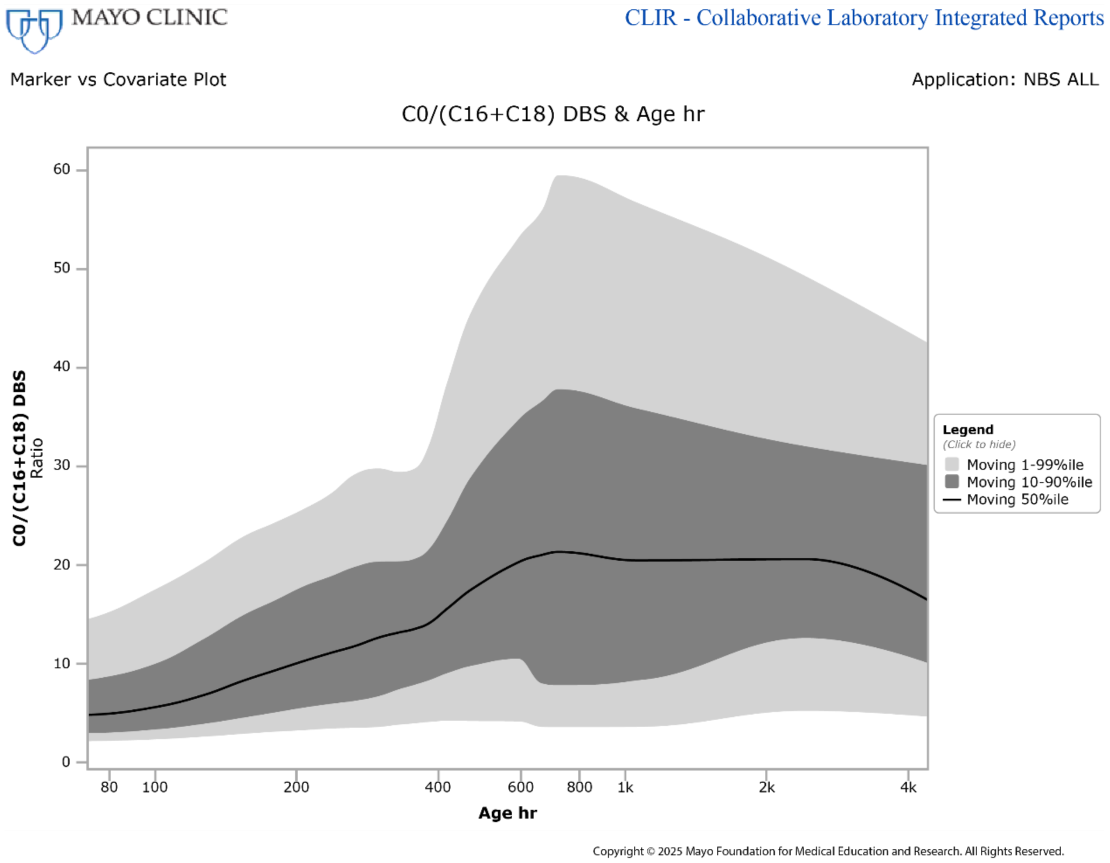

Figure S3. Moving percentiles derived from CLIR, indicating the impact of age on the C0/(C16+C18) ratio in newborn infants. Retrieved from https://clir.mayo.edu/ (accessed on the 17th of June 2025).

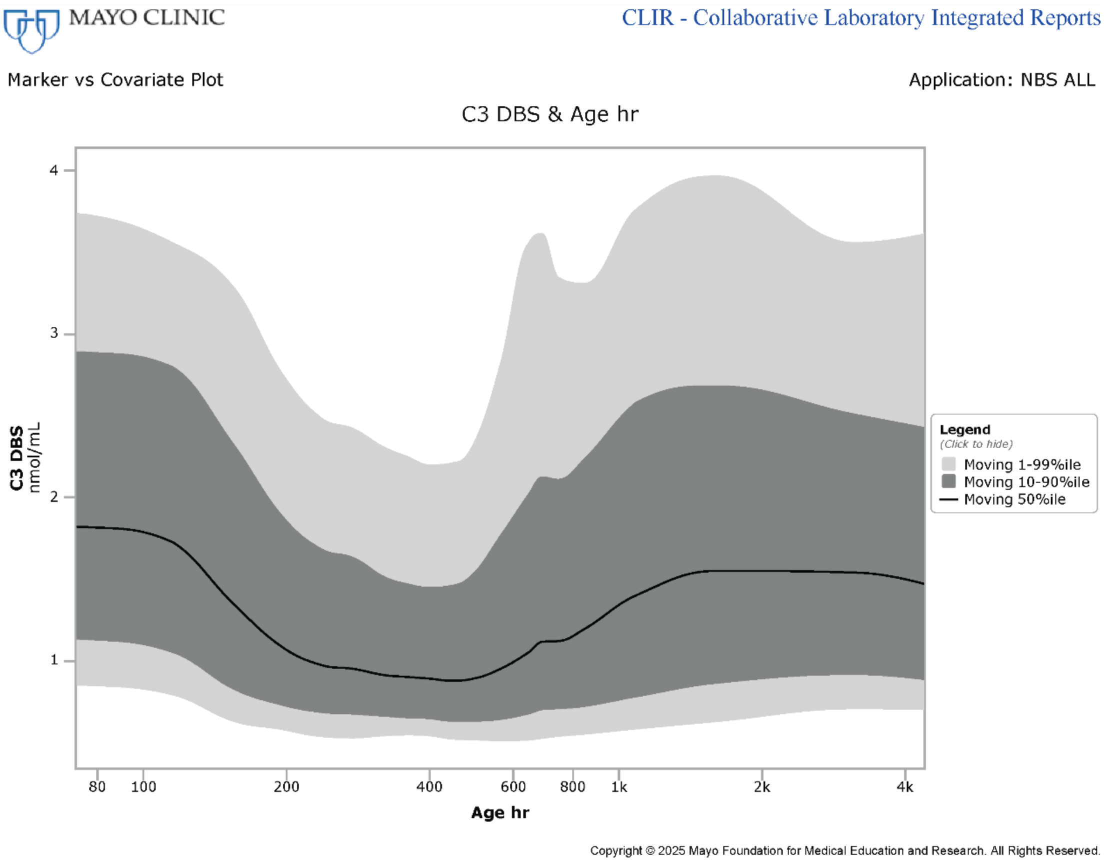

Figure S4 Moving percentiles derived from CLIR, indicating the impact of age on C3 levels (nmol/ml) in newborn infants. Retrieved from https://clir.mayo.edu/ (accessed on the 17th of June 2025).

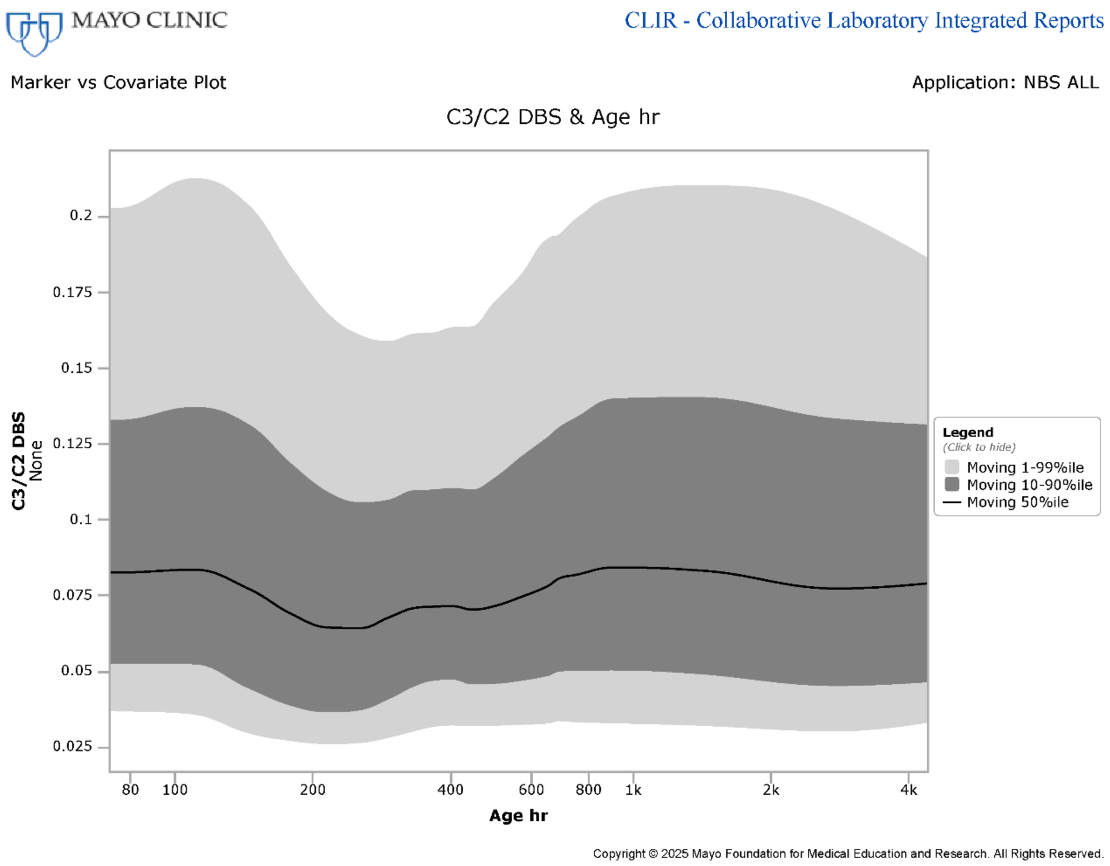

Figure S5. Moving percentiles derived from CLIR, indicating the impact of age on the C3/C2 ratio in newborn infants. Retrieved from https://clir.mayo.edu/ (accessed on the 17th of June 2025).

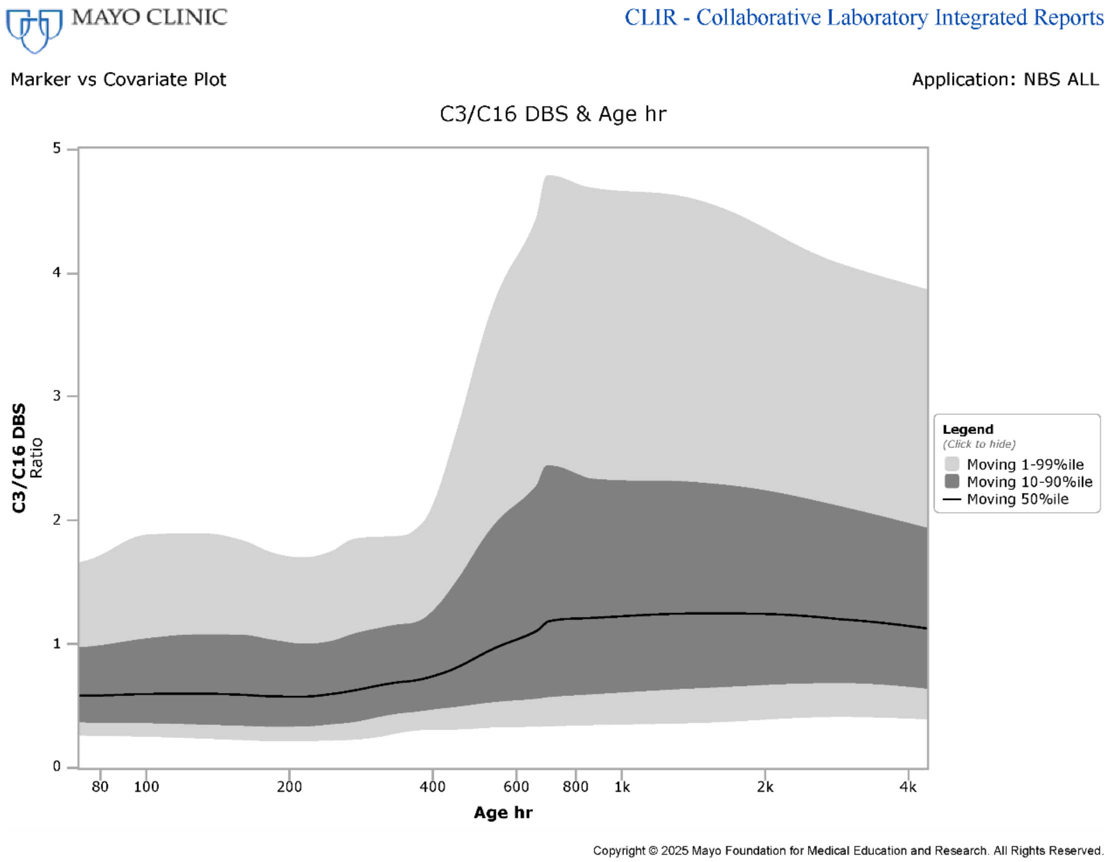

Figure S6. Moving percentiles derived from CLIR, indicating the impact of age on the C3/C16 ratio in newborn infants. Retrieved from https://clir.mayo.edu/ (accessed on the 17th of June 2025).

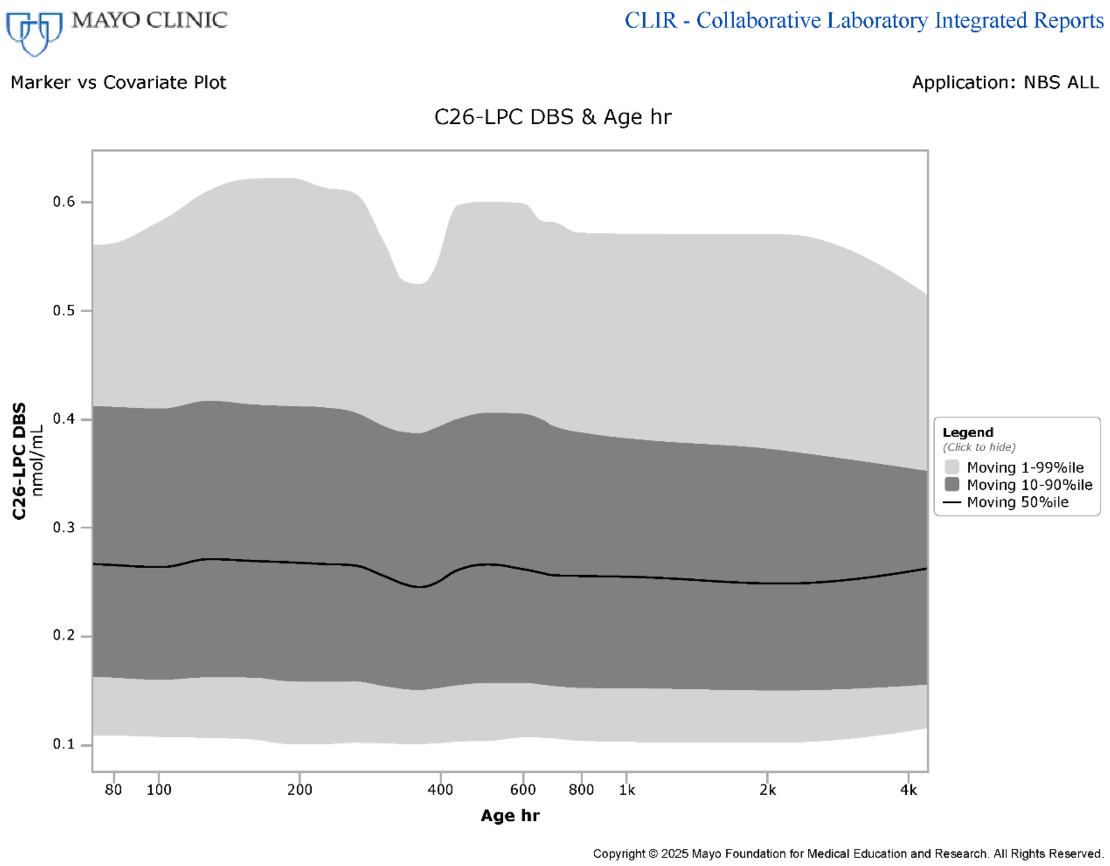

Figure S7. Moving percentiles derived from CLIR, indicating the impact of age on C26:0 LPC levels (nmol/ml) in newborn infants. Retrieved from https://clir.mayo.edu/ (accessed on the 17th of June 2025).

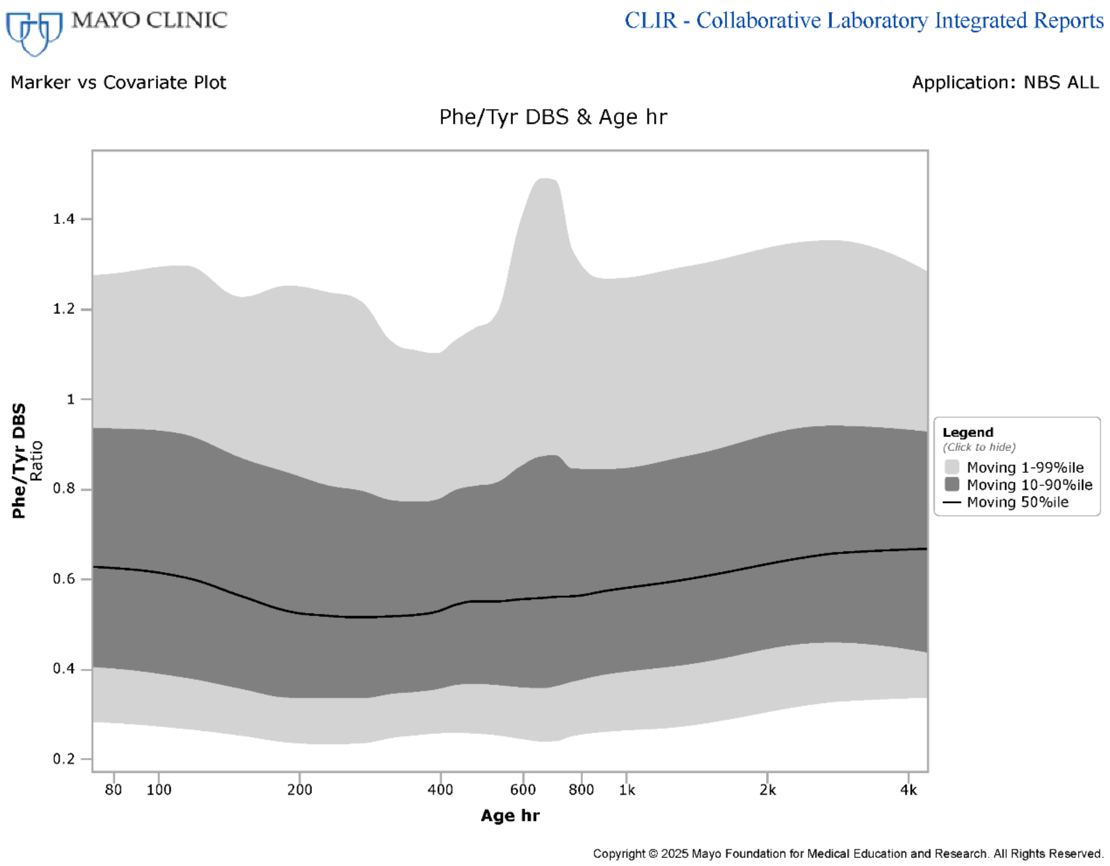

Figure S8. Moving percentiles derived from CLIR, indicating the impact of age on the PHE/TYR ratio in newborn infants. Retrieved from https://clir.mayo.edu/ (accessed on the 17th of June 2025).

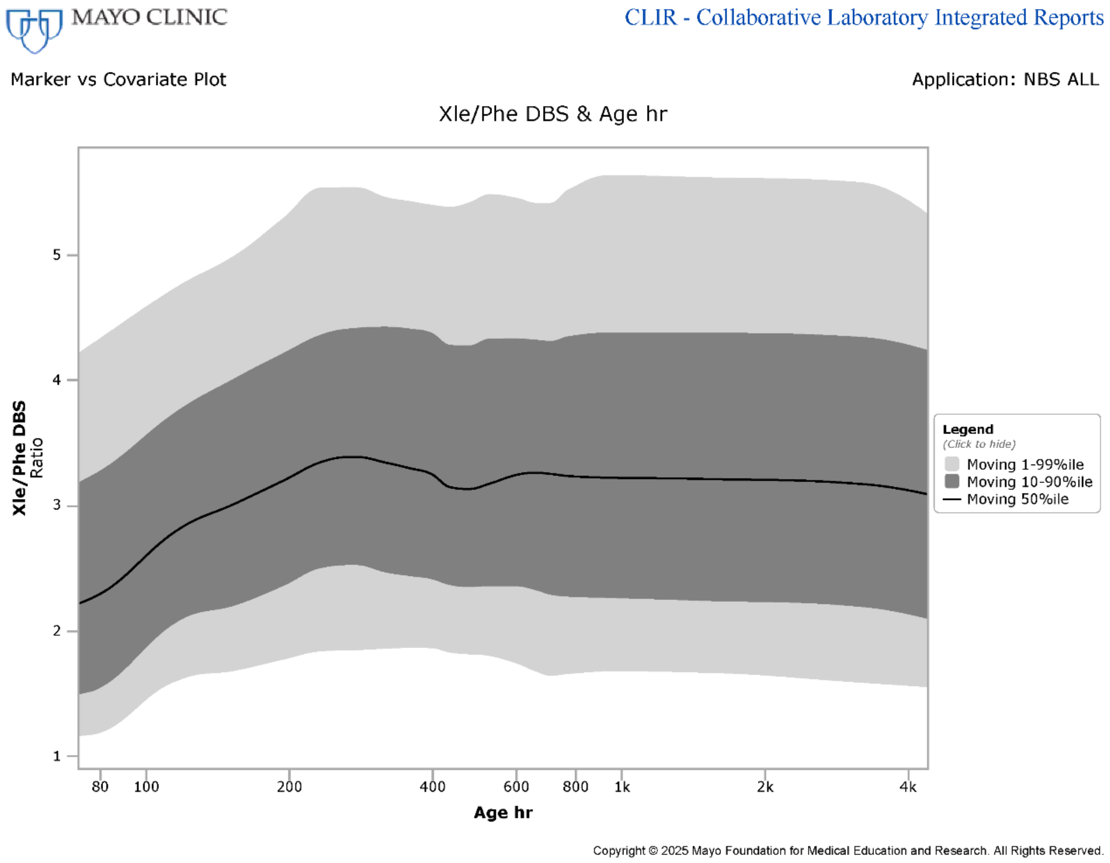

Figure S9. Moving percentiles derived from CLIR, indicating the impact of age on the LEU/PHE ratio in newborn infants. Retrieved from https://clir.mayo.edu/ (accessed on the 17th of June 2025).
